# *Caenorhabditis elegans* muscle Cys-loop receptors as novel targets of terpenoids with potential anthelmintic activity

**DOI:** 10.1371/journal.pntd.0007895

**Published:** 2019-11-25

**Authors:** Guillermina Hernando, Ornella Turani, Cecilia Bouzat

**Affiliations:** Instituto de Investigaciones Bioquímicas de Bahía Blanca, Departamento de Biología, Bioquímica y Farmacia, Universidad Nacional del Sur (UNS)-CONICET, Bahía Blanca, Argentina; University of Pennsylvania, UNITED STATES

## Abstract

The anthelmintic treatment of nematode infections remains the pillar of worm control in both human and veterinary medicine. Since control is threatened by the appearance of drug resistant nematodes, there is a need to develop novel compounds, among which phytochemicals constitute potential anthelmintic agents. *Caenorhabditis elegans* has been pivotal in anthelmintic drug discovery and in revealing mechanisms of drug action and resistance. By using *C*. *elegans*, we here revealed the anthelmintic actions of three plant terpenoids -thymol, carvacrol and eugenol- at the behavioral level. Terpenoids produce a rapid paralysis of worms with a potency rank order carvacrol > thymol > eugenol. In addition to their paralyzing activity, they also inhibit egg hatching, which would, in turn, lead to a broader anthelmintic spectrum of activity. To identify drug targets, we performed an *in vivo* screening of selected strains carrying mutations in receptors involved in worm locomotion for determining resistance to the paralyzing effect of terpenoids. The assays revealed that two Cys-loop receptors with key roles in worm locomotion -Levamisole sensitive nicotinic receptor (L-AChR) and GABA(A) (UNC-49) receptor- are involved in the paralyzing effects of terpenoids. To decipher the mechanism by which terpenoids affect these receptors, we performed electrophysiological studies using a primary culture of *C*. *elegans* L1 muscle cells. Whole cell recordings from L1 cells demonstrated that terpenoids decrease macroscopic responses of L-AChR and UNC-49 receptor to their endogenous agonists, thus acting as inhibitors. Single-channel recordings from L-AChR revealed that terpenoids decrease the frequency of opening events, probably by acting as negative allosteric modulators. The fact that terpenoids act at different receptors may have important advantages regarding efficacy and development of resistance. Thus, our findings give support to the use of terpenoids as either an alternative or a complementary anthelmintic strategy to overcome the ever-increasing resistance of parasites to classical anthelmintic drugs.

## Author summary

Parasitic nematodes (roundworms) are of major significance as human pathogens and have important economic impact worldwide due to considerable losses in livestock and food crops. Drug treatment of nematode infections (anthelmintic drugs) are the pillar of worm control in human and veterinary medicine. Due to the appearance of drug resistant nematodes, there is a need of developing novel drugs, among which phytochemicals, that have environmental sustainability advantages, may constitute potential anthelmintic compounds. As parasitic nematodes are not ideal laboratory animals, the free-living nematode *Caenorhabditis elegans*, which shares many physiological characteristics with parasites and is sensitive to anthelmintic drugs, has emerged as a model organism for anthelmintic drug discovery. We found that three terpenoid compounds present in essential oil of plants–thymol, carvacrol and eugenol–produce rapid paralysis of *C*. *elegans* and inhibit egg hatching, thus mediating both rapid and long-term anthelmintic effects. By testing mutant worms that lack receptor proteins essential for locomotion we identified two different muscle receptors, nicotinic and GABA receptors, as terpenoid targets of the paralyzing effects. Electrophysiological studies from *C*. *elegans* cultured muscle cells demonstrated that terpenoids inhibit the function of these receptors. Thus, by modulating two receptors with key roles in worm motility, these terpenoids emerge as novel anthelmintic compounds.

## Introduction

Parasitic nematodes cause extensive morbidity and mortality in humans and animals and have major economic impact worldwide due to considerable losses in livestock and food crops [1]. Humans themselves are host to different roundworm species, some of which are causative agents in core neglected tropical diseases, such as trichuriasis, ascariasis, hookworm disease, lymphatic filariasis, onchocerciasis, and dracunculiasis [2,3] These human diseases affect billions of people [4]. Also, gastrointestinal nematodes, such as *Haemonchus contortus* and *Ascaris suum*, are one of the main causes of losses in animal productivity worldwide.

Anthelmintic drugs against these parasites act through different mechanisms, and ion channels are one of the main drug targets [5]. In particular, Cys-loop receptors, which are pentameric-ligand gated ion channels, are targets of widely used anthelmintics, such as levamisole, piperazine and ivermectin. Resistance of nematodes to the limited number of anthelmintic drugs available has become a global concern for veterinary and human health. There is therefore an urgent need for concerted efforts to develop novel anthelmintic agents.

The free-living nematode *Caenorhabditis elegans* is a valuable tool for the study of anthelmintic targets because it shares physiological and pharmacological characteristics with parasitic nematodes, it is sensitive to most anthelmintic drugs and it is a useful model organism for drug testing [6,7].

The muscle levamisole-sensitive acetylcholine receptor (L-AChR) and the γ-aminobutyric acid (GABA) type A (UNC-49) receptor are Cys-loop receptors involved in muscle contraction and locomotion of parasitic nematodes and *C*. *elegans*. They are also of clinical relevance as targets of anthelmintic drugs. In this regard, levamisole, which is a full agonist of nematode L-AChRs, produces spastic muscle paralysis and death, and piperazine, which is a GABA receptor agonist, causes flaccid and reversible paralysis of nematode body wall muscle.

Medicinal plants provide an alternative source of potential anthelmintic compounds [8–10]. Monocyclic phenolic compounds, such as thymol, carvacrol and eugenol, are a group of phytochemicals present in essential oils from aromatic plants including thyme (*Thymus vulgaris*), oregano (*Origanum vulgare*) and clove (*Syzygium aromaticum*). These terpenoid phenols have been traditionally recognized for their antinociceptive, local anesthetic, anti-inflammatory and antibacterial actions [11]. There are several reports showing the *in vitro* and *in vivo* effects of terpenoids on parasitic nematodes and their potential as anthelmintic compounds. In the early 1900s, thymol was used for the treatment of ascarids and hookworms in humans [12–14]. *In vitro*, it was shown that thymol and carvacrol have nematocidal activity against *A*. *suum* [15] and that thymol inhibits the motility [16] and egg hatching [8] of *H*. *contortus*. Studies in pigs infected with *A*. *suum* showed that plant-based essential oil blends containing thymol and provided in food reduced infection burdens of helminths, thus showing promise as a daily supplement to reduce infections [14]. Nevertheless, the underlying molecular mechanisms of these anthelmintic actions have not been fully elucidated.

We here used *C*. *elegans* as a model for parasitic nematodes to explore the actions of three terpenoids with anthelmintic activity–thymol, carvacrol and eugenol–and to elucidate the mechanisms and targets by which they induce rapid paralysis. We found that they also inhibit egg hatching, thus mediating both rapid and long-term effects. Our behavioral assays in mutant *C*. *elegans* strains revealed that L-AChRs and UNC-49 (GABA) receptors are main targets involved in the terpenoid effects. Electrophysiological studies from *C*. *elegans* L1 muscle cells were performed to further identify the underlying mechanism. The results suggest that terpenoids inhibit responses to the neurotransmitters by acting as negative allosteric modulators. Thus, by interacting with two types of Cys-loop receptors with antagonic actions at the worm neuromuscular junction, these terpenoids emerge as promising anthelmintic compounds.

## Methods

### *Caenorhabditis elegans* strains

Nematode strains used were: N2: Bristol wild-type; PD4251: *ccIs4251;dpy-20(e1282)*; CB904: *unc-38(e264)*; CB407: *unc-49(e407)*; DA1316: *avr-14(ad1305);avr-15(vu227);glc-1(pk54);* RB918: *acr-16(ok789)*; DA1814: *ser-1(ok345);* MT9668: *mod-1(ok103);* AQ866: *ser-4(ok512)*. All nematode strains were obtained from the *Caenorhabditis* Genetics Center, which is funded by the NIH National Center for Research Resources (NCRR). Nematode strains were maintained at 18–25 °C using freshly prepared Nematode Growth Medium (NGM) petri dishes that had been spread with *Escherichia coli* (OP50) as a source of food [17,18].

### Isolation and culture of *C*. *elegans* muscle cells

Cells were isolated and cultured as described before [17,18]. Briefly, gravid adult nematodes were exposed to an alkaline hypochlorite solution and eggs were treated with 1 unit/ml chitinase. The dissociated embryo cells were filtered and placed on glass coverslips coated with poly-L-Ornithine. Cultures were maintained at 22–24 °C in a humidified incubator in L-15 medium containing 10% fetal bovine serum. Complete differentiation to the various cell types that comprise the newly hatched Larva 1 (L1) was observed within 24 h as described by Christensen *et al*. [19]. Electrophysiological experiments were performed 1–5 days after cell isolation.

### Patch-clamp recordings

Recordings were carried out at 20 °C in the whole-cell configuration for macroscopic currents and in the cell-attached patch configuration for single-channel currents [17,18]. For single-channel recordings, the bath and pipette solutions contained 142 mM KCl, 5.4 mM NaCl, 1.8 mM CaCl_2_, 1.7 mM MgCl_2_, and 10 mM HEPES (pH 7.4). Single-channel currents were recorded using an Axopatch 200 B patch-clamp amplifier (Molecular Devices), digitized at 5 μs intervals, and detected by the half-amplitude threshold criterion using the TAC 4.0.10 program (Bruxton Corporation). Open- and closed-time histograms were plotted using a logarithmic abscissa and a square root ordinate and fitted to the sum of exponential functions by maximum likelihood using TACFit (Bruxton Corporation). To recognize bursts and quantify their durations, a critical closed time (t_crit_) was defined as the point of intersection between the briefest and the succeeding component, and openings separated by closings briefer than this time constitute a burst [20]. Typically, t_crit_ ranged from 0.15 ms to 0.20 ms. Burst duration histograms were well described by the sum of two exponentials, with the briefest duration component corresponding to isolated events and the longest duration component, to bursts. The burst duration was taken from the duration of the slowest component of the burst duration histogram.

Macroscopic currents were recorded in the whole-cell configuration as described before [21]. The pipette solution contained 134 mM KCl, 10 mM EGTA, 1 mM MgCl_2_, and 10 mM HEPES (pH 7.3). The extracellular solution (ECS) contained 140 mM NaCl, 3 mM CaCl_2_, 5 mM KCl, 5 mM MgCl_2_, 11 mM glucose and 5 mM HEPES (pH 7.4). The cell membrane capacitance (Cm) was determined using the software Windows Whole Cell Program (WinWCP; Strathclyde Institute of Pharmacy and Biomedical Sciences, UK) after obtaining the whole-cell configuration. All tested L1 muscle cells exhibited Cm values varying from 2 to 6 pF.

Drugs were obtained from Sigma-Aldrich Co. The stock solutions for ivermectin and terpenoids were prepared in dimethyl sulphoxide (DMSO) and the final DMSO concentration used in all assays was lower than 0.1%.

### Motility assays

Assays were performed with young adult hermaphrodites (2–4 days after hatching) or L1 worms from synchronized plates. Paralysis was determined on fresh agar plates without bacteria and containing the tested drug or the vehicle at room temperature as described before [17,21]. Body paralysis was followed by visual inspection at the indicated time (every 15, 30 or 60 min) and was defined as the lack of complete body movement in response to prodding. For prodding, we used the gentle touch stimulus delivered to the body with an eyebrow hair, avoiding touching the animals too near the tip of the head or tail [22,23]. We evaluated different types of nematode paralysis: flaccid that is mediated by inhibitory stimulation, in which worms appear lengthened, and spastic, which is mediated by L-AChR stimulation and worms appear shorter [17]. Ivermectin produces stationary paralysis, in which worms are paralyzed but retain the capability of responding to prodding by contracting body wall muscle [24]. All assays were carried out by two independent operators and were blinded to the sample identities.

### *C*. *elegans* egg hatching assay

We determined the ability of drugs to inhibit egg hatching according to the following equation: Fraction of unhatched eggs: number of unhatched eggs / (number of hatched larvae + number of unhatched eggs). About 300–500 eggs were placed in an Eppendorf tube containing M9 buffer, M9 and the vehicle DMSO or the tested compound. After an incubation period of 12 h at 22 °C, the content was recovered, washed with M9 buffer and placed into fresh agar plates for counting. The number of unhatched eggs and L1 was counted under a Stereoscopic Zoom Microscope.

### Body length measurement

Synchronized young adult worms were picked to fresh agar plates containing 0.2 mM levamisole, 2 mM thymol, 2 mM carvacrol or 2 mM eugenol and the vehicle DMSO was added into the control plates. Worms (n = 30 per condition) were incubated 2 h at 20 °C. Body length measurement was determined using the free Java ImageJ processing program [25]. At least three independent experiments were carried out.

### Statistics

Experimental data are shown as mean ± SD. Statistical comparisons were done using the Student’s t test, one-way ANOVA followed by Bonferroni’s or Dunn’s Method for multiple comparison post test. A level of p<0.05 was considered significant.

For determining the type of effects of thymol and levamisole combination (synergistic, additive or antagonistic) we used the computer software, CompuSyn [26,27]. Combination index (CI) was calculated from the algorithms for CI using CompuSyn software. CI = 1, < 1 and > 1 indicates additive effect, synergism and antagonism, respectively.

## Results

### Terpenoids paralyze *C*. *elegans* and inhibit egg hatching

**Fig 1:**
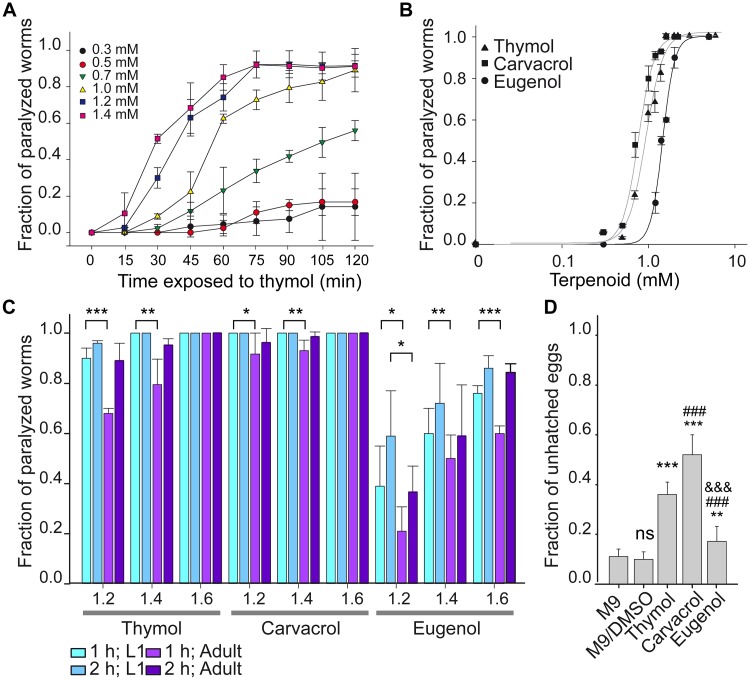
Terpenoids inhibit *C*. *elegans* locomotion as a function of time and concentration. (A) Synchronized adult wild-type worms were placed on agar plates containing increasing thymol concentrations and observed at the indicated time to determine the fraction of paralyzed worms. (B) Dose-response curves for the paralysis exerted by thymol, carvacrol, and eugenol after 1 h exposure. (C) Bar chart showing the fraction of paralyzed worms in the presence of different terpenoids for adult and L1 worms. Synchronized L1 and adult wild-type worms were exposed 1 h and 2 h to the indicated concentration of each terpenoid on agar plates. The results are shown as mean ± SD of at least 5 independent experiments for each condition. At least 30 worms were used for each assay. The symbol * indicates statistically significant differences between L1 and adult worms of each group (1 h or 2 h exposure); p-values for 1 h from left to right were: p = 0.0001; p = 0.0021; p = 0.0361; p = 0.0045; p = 0.0473; p = 0.0052; p = 0.0001 and for 2 h was: p = 0.0383. (D) Bar chart showing the ability of terpenoids to inhibit egg hatching. Fraction of unhatched eggs exposed to M9 buffer or M9/DMSO (0.58%) alone or containing 1.2 mM thymol, carvacrol or eugenol. Results are shown as mean ± SD of 3 different assays for each condition. The symbol * indicates differences with control group, # indicates differences with thymol treatment, & indicates differences with carvacrol treatment, ns: not statistically significant. Two symbols indicate p<0.01 (p = 0.00352) and three symbols indicate p<0.001 (*p-values from left to right were: p = 1.465E-12 and p = 4.254E-12; #p-values from left to right were: p = 0.0000000157 and p = 0.0000000107; and &p = 0.000000000209).

We first explored how terpenoids rapidly affect the whole organism by evaluating the response of young adult *C*. *elegans* to thymol exposure as a function of time and concentration (Fig 1A). To this end, synchronized young adult wild-type worms were placed on agar plates containing thymol at different concentrations, and the fraction of paralyzed worms was determined at different times. A clear time-dependent paralysis was observed (Fig 1A). 100% of worms were paralyzed by thymol concentrations above 1.6 mM after 1 h exposure (Fig 1B) and about 90% were paralyzed by concentrations above 1 mM after 2 h exposure (Fig 1). The EC50 values determined for the paralyzing activity were 0.95 ± 0.03 mM for 1 h exposure (Fig 1B) and 0.70 ± 0.02 mM for 2 h exposure (n ≥ 5 independent experiments for each condition).

To compare the potency of monoterpenoids we also tested carvacrol, a thymol phenol isomer, and eugenol, a phenylpropanoid molecule (Fig 1B). The three terpenoids showed a concentration-dependent inhibition of motility; the order of the paralyzing potency was carvacrol (EC50 = 0.80 ± 0.13 mM for 1 h and 0.60 ± 0.023 mM for 2 h) > thymol >eugenol (EC50 = 1.45 ± 0.02 mM for 1 h and 1.10 ± 0.02 mM for 2 h).

Although paralyzing effects of terpenoids have been reported before in parasites and *C*. *elegans* [15,28], the features of terpenoid-induced paralysis have not been described. Thus, we measured body length of *C*. *elegans* exposed 2 h to 2 mM thymol, 2 mM carvacrol, 2 mM eugenol, 0.2 mM levamisole or DMSO (0.8%) on agar plates. Levamisole was used as a control since it produces spastic paralysis. As expected, levamisole reduced body length. The relative body length (RBL) in the presence of levamisole compared to that in control agar plates was 0.93 ± 0.05 (p<0.05). In contrast, visual inspection and measurement of worm length revealed that terpenoids do not change significantly body length. The RBL values were 1 ± 0.08 for thymol, 1 ± 0.09 for carvacrol, 1 ± 0.1 for eugenol, and 1 ± 0.06 for the vehicle DMSO (p>0.15). Thus, terpenoids produced neither flaccid nor spastic paralysis of *C*. *elegans* worms.

With the aim of comparing terpenoid sensitivities among developmental stages, we exposed the first larval stage (L1) and young adult worms to the tested terpenoids. 1 h and 2 h exposure of L1 worms to 1.2 mM, 1.4 mM and 1.6 mM thymol, carvacrol or eugenol significantly affected worm locomotion (Fig 1C). There were no paralyzed worms in the control plates with DMSO at both stages, and all worms were moving at the beginning of the assay. L1 worms were more sensitive to terpenoids during the first hour than adult worms (n = 5 experiments for each condition).

To determine if terpenoids affect egg-hatching, a property related to anthelmintic capability, we performed the egg-hatching assay with modifications for *C*. *elegans* [29]. *C*. *elegans* eggs were exposed to M9, M9/DMSO or M9 containing 1.2 mM thymol, carvacrol or eugenol for 12 h and the fraction of unhatched eggs was determined. We showed that all terpenoids inhibit egg-hatching (Fig 1D). The egg-hatching inhibition effect was stronger for carvacrol than for thymol and eugenol, in agreement with the potency for nematode muscle paralysis (Fig 1D). The effect appeared to be irreversible since eggs treated for 12 h with terpenoids at the corresponding ~EC50 concentrations did not show hatching after being washed with M9 buffer, seeded into fresh agar plates (lacking the compounds), and incubated for additional 12 h.

### Deciphering the molecular targets mediating terpenoid paralysis: Mutants lacking L-AChR or UNC-49 receptors are partially resistant

**Fig 2:**
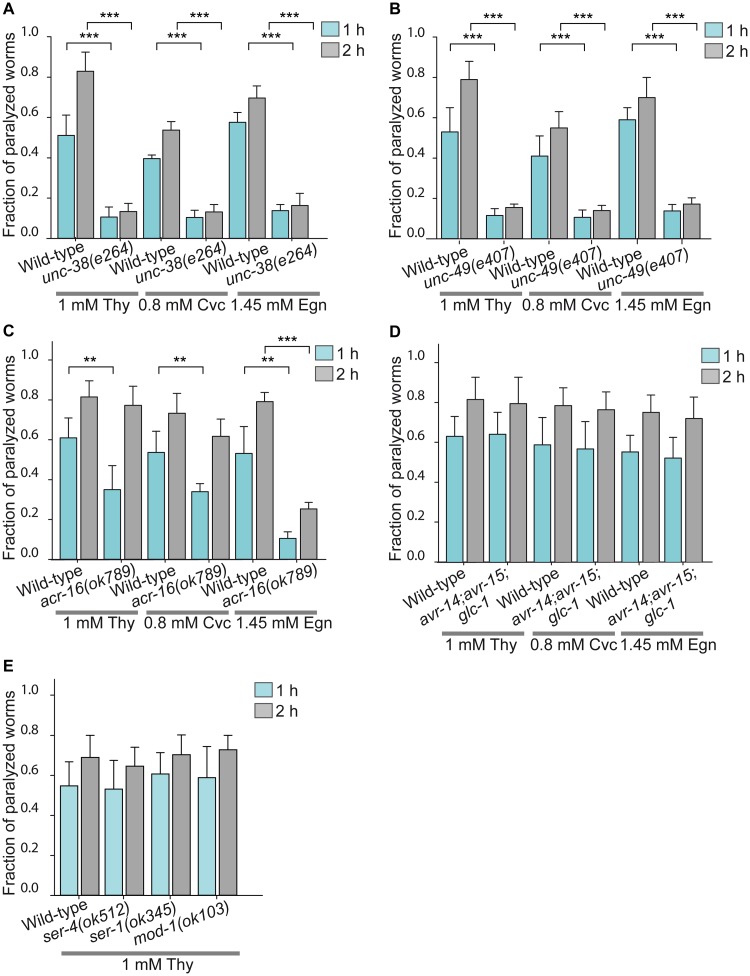
Deciphering molecular targets mediating terpenoid effects by using mutant strains. Wild-type and mutant adult worms were placed on agar plates containing thymol, carvacrol or eugenol at EC50 concentrations calculated for the wild-type strain and the fraction of paralyzed worms was measured for each condition. Each point represents the average of ≥ 5 experiments, n = 30 worms, error bars = SD. *C. elegans* mutant strains are: (A) *unc-38(e264)*; corresponds to mutants of UNC-38 subunit, which lack functional L-AChRs; (B) *unc-49(e407)*, corresponds to null mutants of UNC-49B subunit which lack functional UNC-49 receptor; (C) *acr-16(ok789)* that lacks the homopentameric nicotine-sensitive AChR (N-AChR or ACR-16); (D) The triple mutant strain of glutamate-gated chloride channel receptor (GluClRs) subunits, *avr-14(ad1305);avr-15(vu227);glc-1(pk54)*, which has been shown to exhibit high resistance to ivermectin; (E) *ser-4(ok512)* that lacks a metabotropic serotonin receptor (SER-4); *ser-1(ok345)* that lacks a metabotropic serotonin receptor (SER-1), and *mod-1(ok103)* that lacks the serotonin-activated chloride channel (MOD-1). Results are shown as mean ± SD. The symbol * indicates statistically significant differences between wild-type and mutant worms of the same group (1 h or 2 h exposure). **p<0.01 (p-values from left to right were: p = 0.0047, p = 0.0057 and p = 0.008) and ***p<0.001 (p≤0.0001).

To determine if Cys-loop receptors are involved in the rapid paralyzing effects of terpenoids, we explored the actions of thymol, carvacrol and eugenol on mutant strains. The rationale for this assay is that the absence of the drug target will cause resistance to the drug. Fig 2 shows the fraction of paralyzed worms exposed to 1 mM thymol, 0.8 mM carvacrol and 1.45 mM eugenol, which correspond to the EC50 concentrations determined for wild-type worms.

We first tested mutant strains lacking the main Cys-loop receptors involved in muscle contraction, L-AChR (homologous to the mammalian muscle nAChRs [30]) and UNC-49 receptor (homologous to the mammalian GABA(A) receptor [31]). In the *unc-38(e264)* mutant strain, which lacks functional L-AChRs due to the absence of the essential UNC-38 subunit, the fraction of paralyzed worms exposed to terpenoids at EC50 concentrations was notably reduced with respect to wild-type worms; less than 20% of mutant worms were paralyzed at 1 h or 2 h exposure (Fig 2A). The increase of terpenoid concentrations (1.2 mM thymol, 1.2 mM carvacrol or 1.6 mM eugenol) increased the fraction of paralyzed worms but the effects were also statistically significantly different with respect to wild-type worms (S1 Fig). The *unc-38(e264)* mutant was 2.5 ± 1.1 and 1.5 ± 0.7 times less sensitive than the wild-type after 1 h and 2 h exposure to 1.2 mM thymol, respectively (S1 Fig). As in wild-type worms, exposure for 2 h to 2 mM terpenoids led to ~100% paralysis, indicating that the mutant is partially resistant to the compounds. The percentage of paralyzed worms at 2 mM were: For thymol: 0.996 ± 0.0089 (wild-type) and 0.970 ± 0.042 (*unc-38(e264)*) (p = 0.2), for carvacrol: 1.00 ± 0.004 (wild-type) and 0.960 ± 0.043 (*unc-38(e264)*) (p = 0.17), for eugenol: 0.968 ± 0.0041 (wild-type) and 0.966 ± 0.045 (*unc-38(e264)*) (p = 0.9).

In the *unc-49(e407)* mutant strain, which lacks UNC-49 receptors, the fraction of paralyzed worms exposed to terpenoids at EC50 concentrations was reduced with respect to wild-type worms; less than 20% of mutant worms were paralyzed at 1 h or 2 h exposure (Fig 2B). As observed for the *unc-38* mutant strain, the paralysis of the *unc-49(e407)* mutant strain was concentration dependent since the fraction of paralyzed worms increased by increasing terpenoid concentrations (1.2 mM thymol, 1.2 mM carvacrol or 1.6 mM eugenol) (S1 Fig). The mutants were 1.97 ± 0.7 and 1.35 ± 0.15 times less sensitive to 1.2 mM thymol than wild-type worms in the first and second hour, respectively (S1 Fig). Also, ~100% of *unc-49(e407)* worms were paralysis when exposed for 2 h at 2 mM terpenoid. The fraction of paralyzed mutant worms at 2 mM were: 0.97 ± 0.05 (p = 0.3), 0.972 ± 0.027 (p = 0.09), and 0.95 ± 0.057 (p = 0.5) for thymol, carvacrol and eugenol, respectively.

The results indicate that L-AChR and UNC-49 receptors are involved in terpenoid-induced paralysis.

We also tested the mutant strain *acr-16(ok789)* that lacks the homopentameric nicotine-sensitive AChR (N-AChR or ACR-16, homologous to α7 nAChR mammalian receptor [32]), which is also present in the nematode muscle. The exposure of mutant worms to terpenoids at concentrations corresponding to the EC50 values determined for wild-type worms showed that the fraction of paralyzed worms was slightly, but significantly, lower than that of wild-type worms (Fig 2C). With respect to wild-type worms, there were no statistically significant differences in the fraction of paralyzed worms at 1.2 mM thymol (S1 Fig) and 2 mM thymol (0.98 ± 0.04, p = 0.4). Thus, this receptor may also play a role in the paralysis caused by terpenoids, although the effect is not as marked as that mediated by GABA and L-AChR receptors.

Likewise, we tested the contribution of other receptors associated with anthelmintic action or worm locomotion to the terpenoid paralysis. The triple mutant strain of glutamate-gated chloride channel receptors (GluClRs) subunits, *avr-14(ad1305);avr-15(vu227);glc-1(pk54)*, which has been shown to exhibit high resistance to ivermectin [33], showed no statistically significant differences with respect to the wild-type strain in the sensitivity to terpenoids at concentrations corresponding to the EC50 values determined for wild-type worms (Fig 2D) or at 1.2 mM thymol (Fig 1S).

We also tested mutant strains of serotonin receptors in *C*. *elegans* as the serotonin system controls several behaviors in nematodes: *ser-4(ok512)*, which lacks the metabotropic serotonin receptor SER-4, *ser-1(ok345)*, which lacks the metabotropic serotonin receptor SER-1, and *mod-1(ok103)* that lacks the serotonin-activated chloride channel (MOD-1). The mammalian orthologs for these receptors are 5-HT1, 5-HT2 and glycine receptors, respectively [34–36]. No significant differences on the fraction of paralyzed worms were found between wild-type and mutant strains for thymol concentrations 1 mM (p>0.20, Fig 2E) and 1.2 mM (p>0.30, S1 Fig), indicating that these receptors are not involved in the paralysis induced by thymol.

### Combined chemotherapy with thymol and classical anthelmintic drugs

In anthelmintic therapy, the combination of drugs is a strategy used to reduce acquisition of resistance. We used thymol as the prototype drug on agar plates in combination with currently used anthelmintics. We performed paralysis assays on agar plates in the presence of thymol alone and in combination with levamisole that activates the L-AChR, piperazine that activates UNC-49 receptors and elicits flaccid paralysis, or ivermectin that activates GluClR and also acts on GABA receptors [21].

**Fig 3:**
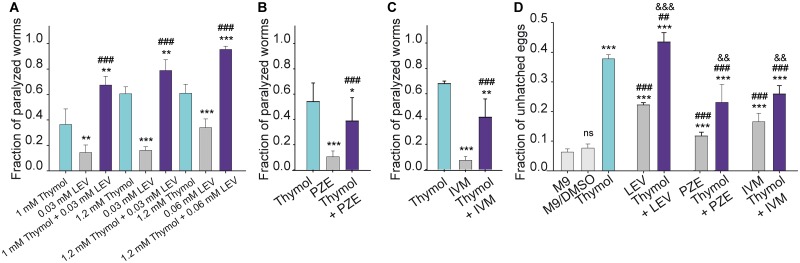
Thymol and its combination with classical anthelmintics. Bar chart showing the fraction of paralyzed worms after 1 h-exposure on agar plates to 1 and 1.2 mM thymol, 0.03 and 0.06 mM levamisole (LEV) and the combinations 1 mM thymol and 0.03 mM levamisole, 1.2 mM thymol and 0.03 mM levamisole and 1.2 mM thymol and 0.06 mM levamisole (A), 1.2 mM thymol or 30 mM piperazine (PZE) and its combination (B), 1.2 mM thymol or 0.3 mM ivermectin (IVM) and its combination (C). The results are shown as mean ± SD of at least 5 independent experiments for each condition. At least 30 worms were used for each assay. (D) Bar chart showing the fraction of unhatched eggs exposed to M9, M9/DMSO (0.58%), 1.2 thymol, 0.2 mM levamisole (LEV), 25 mM piperazine (PZE), 0.02 mM ivermectin (IVM) and the combinations. Results are shown as mean ± SD of 3 different assays for each condition. The symbol * indicates differences with control group (M9), ^#^indicates differences with thymol treatment, ^&^indicates differences with the anthelmintic agent and ns: not statistically significant. One symbol indicates p<0.05 (p = 0.048); two symbols indicate p<0.01 (p-values from left to right were: p = 0.0063; p = 0.00113; p = 0.00359; p = 0.0036; p = 0.0037; p = 0.0023 and p = 0.0015) and three symbols indicate p<0.001 (p≤0.0001).

After 1 h exposure, ~15% of worms were paralyzed in the presence of 0.03 mM levamisole. The combination of 0.03 mM levamisole with 1.2 mM thymol resulted in ~80% of paralyzed animals (Fig 3A). We performed a more detailed analysis for the thymol/levamisole combination to determine if the effect is additive or synergistic. In the same set of experiments, we measured the fraction of paralyzed worms at different concentrations of thymol (0.3 mM-1.4 mM) and levamisole (0.005 mM-0.06 mM) (Fig 3A and S2 Fig). We also measured in the same set of experiments the paralysis with three different drug combinations (0.03 mM levamisole/ 1 mM thymol; 0.03 mM levamisole/ 1.2 mM thymol and 0.06 mM levamisole/ 1.2 mM thymol). The results showed higher paralyzing effect for each respective combination than that exerted by each individual drug at the same concentration. The calculated CI values for each combination, determined by CompuSyn software, were 0.9, 0.86 and 0.53, respectively, indicating that the action can be considered slightly synergic (S2 Fig) [26,37].

For piperazine, high concentrations of the drug on agar plates are required to produce paralysis of worms [21]. Exposure of worms to 30 mM piperazine for 1 h produced ~10% of paralysis. The combination of 30 mM piperazine with 1.2 mM thymol resulted in ~38% paralyzed worms in 1 h (Fig 3B), indicating that the effect was lower than that exerted by thymol alone.

The macrocyclic lactones have a potent, broad antiparasitic spectrum at low dose levels. After 1 h exposure on agar plates containing 0.3 mM ivermectin, ~7% of worms were paralyzed and most of the worms were stationary but they still responded to prodding as reported previously ([21] and Fig 3C). The combination of 0.3 mM ivermectin with 1.2 mM thymol resulted in ~41% paralysis (Fig 3C), which is lower than that exerted by thymol alone.

To determine if combinations of terpenoids and classical anthelmintics also affect egg-hatching, we performed the egg-hatching assay for *C*. *elegans* eggs exposed to M9, M9/DMSO or M9 containing thymol, thymol-levamisole, thymol-piperazine or thymol-ivermectin for 12 h (Fig 3D). We showed that all combinations inhibit egg hatching. However, compared to the effect exerted by thymol alone, the effect of the combination was slightly higher for thymol/levamisole and lower for thymol/piperazine and thymol/ivermectin.

### Terpenoids inhibit macroscopic responses of muscle L-AChR and UNC-49 receptors

To unequivocally confirm that the main muscle Cys-loop receptors involved in muscle contraction -L-AChR and UNC-49 receptors- are modulated by terpenoids, we recorded macroscopic responses from L1 muscle cells elicited by ACh or GABA (0.5–10 mM) in the whole-cell configuration at -70 mV. We have previously determined that currents elicited by ACh or GABA in L1 cells correspond to L-AChR and UNC-49 receptors, respectively [17,21]. No currents were elicited by application of 0.1 mM thymol, carvacrol or eugenol in the absence of agonists, indicating that, at the tested concentrations, terpenoids were not capable of activating L-AChR or UNC-49 receptors (n = 12 cells).

**Fig 4:**
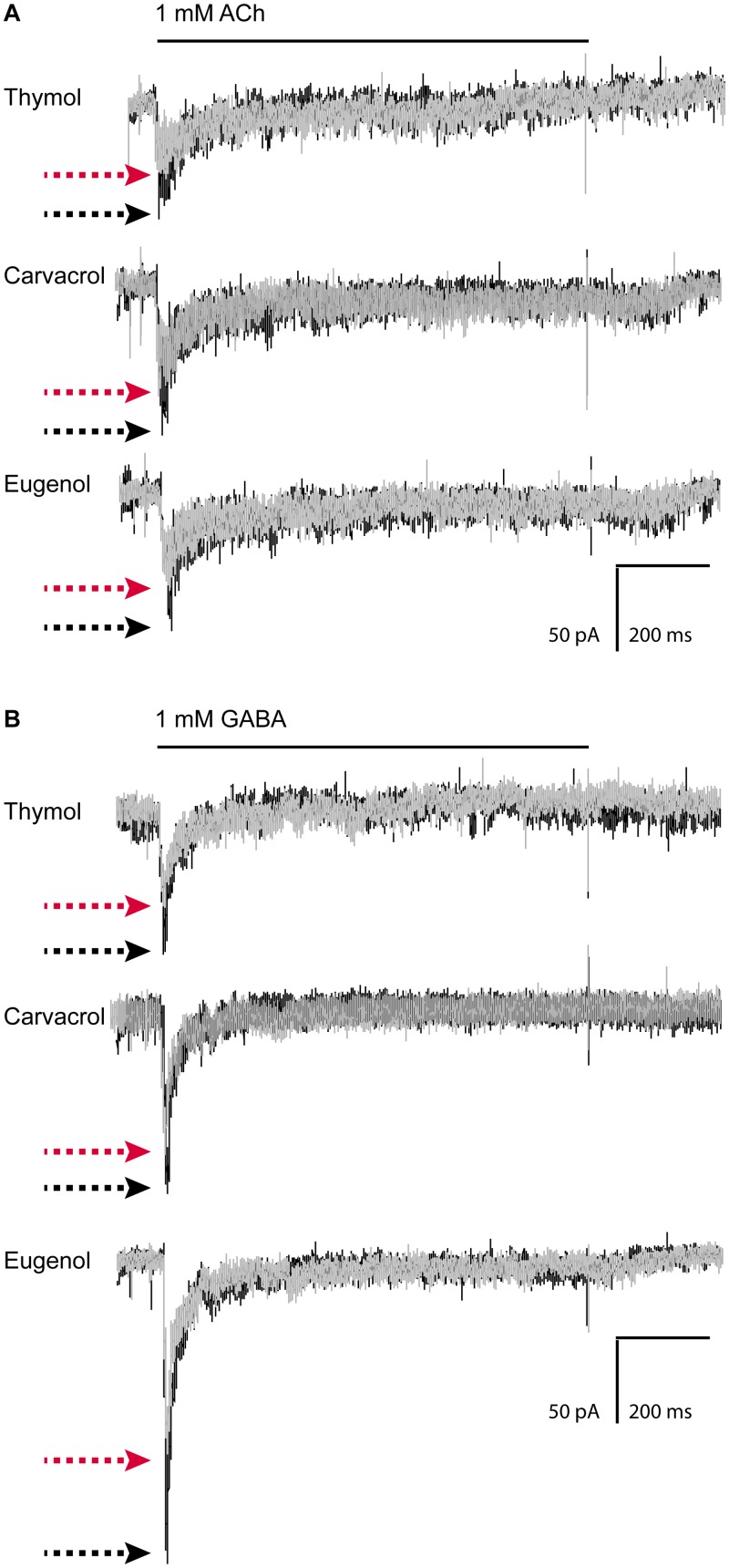
Macroscopic current recordings from L1 muscle cells show that thymol is an inhibitor of L-AChR and UNC-49 receptor. Typical whole-cell traces from L1 cultured muscle cells (PD4251 strain) elicited by a 1 s-pulse of ACh (A) or GABA (B) in the presence or absence of terpenoids. Pipette potential: -70 mV. Each set of currents (control and treated) corresponds to a single cell and each trace represents the average of 2–4 applications of agonist in both control and treated conditions. Whole-cell currents activated by 1 mM ACh or 1 mM GABA were recorded before (black traces) and after pre-exposure to 0.1 mM thymol, 0.1 mM carvacrol or 0.1 mM eugenol, for 1 min (grey traces). The black line shows the time of exposure to the agonist. For clarity, the peaks are indicated by black (control) and red (preincubated with terpenoids) arrows.

After 1 min pre-exposure of L1 cells to 0.1 mM of each terpenoid, the peak currents elicited by ACh or GABA were reduced to 66 ± 10% and 78 ± 9% in the presence of thymol, 56 ± 24% and 54 ± 32% in the presence of carvacrol and 75 ± 19% and 73 ± 16% in the presence of eugenol, respectively (n = 5 cells per condition, Fig 4). The control peak currents were almost fully recovered after 1-min wash with ECS solution. For L-AChR and UNC-49 receptor responses, the recovered currents were 92 ± 13% and 90 ± 16% for thymol, 85 ± 15% and 86 ± 14% for carvacrol; and 86 ± 20% and 98 ± 16% for eugenol, respectively. These results indicate that receptor inhibition is reversible.

Higher terpenoid concentrations (> 0.1 mM) produced membrane instability and could not be studied. Also, the control with DMSO did not produce statistically significant reduction of peak currents (95 ± 6% peak current respect to the control).

Thus, we conclude that thymol, carvacrol and eugenol act as inhibitors of L-AChR and UNC-49 receptors from *C*. *elegans* L1 muscle cells.

### Terpenoids decrease single-channel activity of L-AChRs elicited by ACh and levamisole

**Fig 5:**
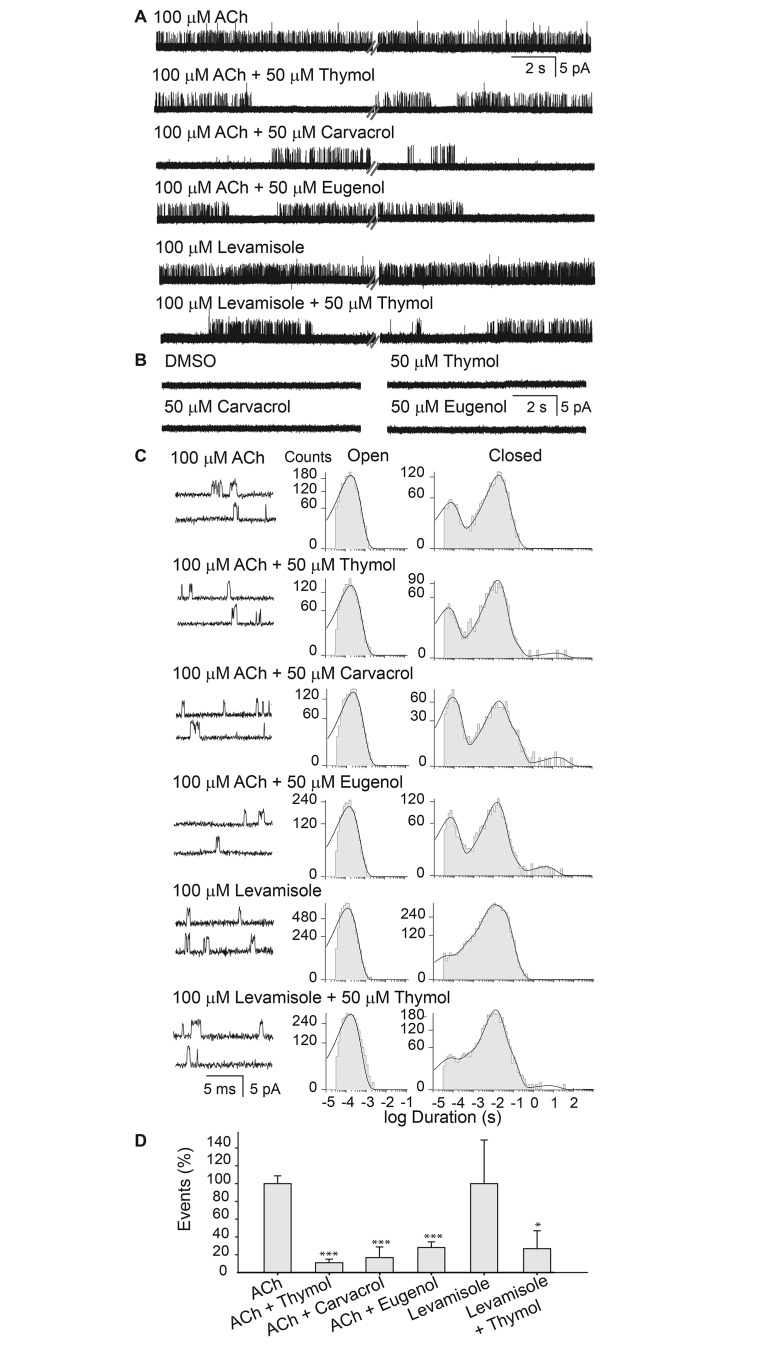
Terpenoids decrease single-channel activity of L-AChRs elicited by ACh. (A) Single channels activated by 100 μM ACh or 100 μM levamisole were recorded in the absence and presence of 50 μM terpenoids in the pipette solution. For each condition, the two different traces, separated by dashes, correspond to the recording during the first (left) and third minute (right). Channel openings are shown as upward deflections. Pipette potential: 100 mV. Filter: 9 kHz. (B) Representative traces of single-channel activity in the presence of DMSO (vehicle) or terpenoids in the pipette solution (in the absence of ACh). (C) Representative traces and open and closed time histograms of channels recorded as described in panel A. For each condition, the trace on the top corresponds to the first min of recording and the one on the bottom, to the third minute of recording. (D) Bar chart showing the comparison of the mean number of opening events ± SD in 1 minute of recording as in panel A. The symbol * indicates statistically significant differences respect to the corresponding control group (ACh or Levamisole).*p<0.05 (p = 0.016) and ***p<0.001 (p-values from left to right: p = 0.000708; p = 0.000646 p = 0.0000535).

To gain further insights into terpenoid action, we recorded single-channel currents from cell-attached patches in L1 muscle cells. Single channels of L-AChRs activated by 1–100 μM ACh or levamisole are readily detected from L1 muscle cells [17,21]. We have previously determined that the detected channel activity is originated from L-AChRs and not from N-AChRs [17,18]. Channel activity recorded at 100 μM ACh or levamisole showed openings of ~3.5 pA (n = 10) at 100 mV pipette potential that appeared mainly isolated or in short bursts formed by two or three successive opening events (Fig 5).

In the presence of terpenoids together with 100 μM ACh in the pipette solution, reduced single-channel activity was observed in all recordings with respect to the controls with ACh alone (Fig 5A). Similar results were observed for 100 μM levamisole and 50 μM thymol in the pipette solution (Fig 5A). As a control, we verified that 0.1% DMSO did not produce any change in single-channel activity (n = 5). We also verified that terpenoids (1, 50 and 100 μM) did not elicit single-channel activity in the absence of ACh (Fig 5B).

We analyzed single-channel currents activated by 100 μM ACh to determine how terpenoids affected channel properties. Activation occurred as isolated events or in bursts of a few openings in quick succession [17]. Open time histograms were fitted by one exponential component, and the mean open times were similar between the control and in the presence of terpenoids in the pipette solution (~0.2 ms, Table 1 and Fig 5C). We found that the mean burst duration (~0.3 ms), obtained from the open time histograms constructed with a critical time of 0.2 ms, did not change significantly in the presence of terpenoids (Table 1). Closed-time distributions of L-AChRs in the absence of terpenoids were well described by the sum of two main components. No statistically significant differences for the duration of the briefest closed components were found in the presence of terpenoids (Table 1). With respect to the second main closed component, there was a trend to longer mean closed durations in the presence of terpenoids, but due to the intrinsic variability of the system, they were only significantly different in the presence of 100 μM carvacrol compared to the control condition (p = 0.01, Table 1). Interestingly, a new minor long-duration component in the second range was present in all recordings in the presence of terpenoids (Fig 5C), indicating the existence of prolonged dwell times in the closed state with respect to control recordings. The duration of this component among different patches was variable since each condition required a different cell patch. However, the mean duration of this closed time increased from 7.1 ± 3.9 s to 23.9 ± 12 s at 1 and 100 μM thymol, respectively (p = 0.03), and from 3.9 ± 1.7 s to 7.9 ± 2.5 s for 1 μM and 100 μM carvacrol, respectively (p = 0.046), indicating a concentration-dependent effect.

**Table 1:** Single-channel properties of L-AChRs activated by 10 μM ACh, 100 μM ACh or 100 μM levamisole in the absence and presence of terpenoids.

Agonist	Treatment	O_1_ (ms) (area)	C_1_ (ms) (area)	C_2_ (ms) (area)	Burst (ms)	n
100 μM ACh	DMSO control	0.213 ± 0.022 (1 ± 0)	0.063 ± 0.008 (0.281 ± 0.025)	19.2 ± 3.114 (0.705 ± 0.021)	0.30 ± 0.03	6
	1 μM Thymol	0.210 ± 0.018 (1 ± 0)	0.077 ± 0.004 (0.551 ± 0.044)	18.667 ± 6.429 (0.385 ± 0.014)	0.46 ± 0.07	5
	1 μM Carvacrol	0.198 ± 0.028 (1 ± 0)	0.08 ± 0.008 (0.483 ± 0.089)	23 ± 1.732 (0.482 ± 0.057)	0.39±0.11	5
	1 μM Eugenol	0.239 ± 0.051 (1 ± 0)	0.069 ± 0.014 (0.464 ± 0.153)	16.030 ± 7.839 (0.487 ± 0.082)	0.55 ± 0.19	5
	50 μM Thymol	0.181 ± 0.021 (1 ± 0)	0.066 ± 0.012 (0.236 ± 0.036)	18.6 ± 3.318 (0.614 ± 0.107)	0.24 ± 0.03	5
	50 μM Carvacrol	0.199 ± 0.014 (1 ± 0)	0.087 ± 0.004 (0.391±0.084)	23 ± 5.196 (0.544 ± 0.129)	0.36 ± 0.05	5
	50 μM Eugenol	0.153 ± 0.005 (1 ± 0)	0.067 ± 0.01 (0.349 ± 0.043)	17.950 ± 4.132 (0.623 ± 0.046)	0.24 ± 0.02	5
	100 μM Thymol	0.266 ± 0.056 (1 ± 0)	0.06 ± 0.011 (0.348 ± 0.105)	32.143 ± 21.019 (0.593 ± 0.112)	0.43 ± 0.13	7
	100μM Carvacrol	0.207 ± 0.05 (1 ± 0)	0.066 ± 0.008 (0.350 ± 0.148)	31.533 ± 10.651 (0.488 ± 0.105)	0.34 ± 0.15	5
100 μM Levamisole	DMSO control	0.250 ± 0.075 (1 ± 0)	0.047 ± 0.026 (0.096 ± 0.049)	55.2 ± 19.96 (0.471 ± 0.109)	0.27 ± 0.1	5
	50 μM Thymol	0.328 ± 0.153 (1 ± 0)	0.073 ± 0.017 (0.150 ± 0.142)	106 ± 83 (0.643 ± 0.236)	0.45 ± 0.31	5
10 μM ACh	DMSO control	0.307 ± 0.056 (1 ± 0)	0.051 ± 0.021 (0.215 ± 0.16)	43.75 ± 23.977 (0.731 ± 0.162)	0.52 ± 0.35	6
	50 μM Thymol	0.304 ± 0.07 (1 ± 0)	0.072 ± 0.014 (0.154 ± 0.085)	48.167 ± 18.335 (0.772 ± 0.098)	0.39 ± 0.13	7

Single channels were recorded from L1 muscle cells at a holding potential of 100 mV. ACh channels corresponding to L-AChRs were recorded in the presence of ACh or levamisole with and without terpenoids in the pipette solution. The mean durations of the open component (O_1_) and closed components (C_1_ and C_2_) and mean burst durations were obtained from the corresponding histograms. Data are shown as mean ± SD and the number of recordings for each condition is indicated (n).

Thymol did not affect single-channel conductance. The relationship between the amplitude of single channels and pipette potential revealed that L-AChRs activated by ACh in presence of thymol exhibit an inward conductance of 32 ± 0.9 pS, similar to that observed in the presence of ACh alone (33 ± 0.9 pS, p = 0.12). Thus, the decrease in the amplitude of macroscopic currents cannot be attributed to a decrease in single-channel amplitude.

The main change in the single-channel pattern due to the presence of terpenoids was evidenced as a reduced frequency of channel opening, which was clearly detected from visual inspection of the recordings (Fig 5A). In the presence of 100 μM ACh or levamisole, the number of events remained constant during the course of the recording (Fig 5A and [17]). However, with the addition of terpenoids (1, 50 or 100 μM), channel activity pattern appeared as alternating silent and active periods. Similar changes were detected for 100 μM levamisole in the presence of 50 μM thymol (Fig 5A). To quantify the decrease in the frequency of channel opening, we measured the number of opening events in 3–5 min of recording and compared the number of openings per minute in the presence of terpenoids to control recordings in the absence of terpenoids (Fig 5D). As shown in the figure, terpenoids exerted a marked, statistically significant decrease of the number of opening events that was not dependent on the type of agonist.

Single-channel activity at low ACh concentrations (10 μM) also occurs as isolated openings or as short bursts of openings in quick succession. Open time histograms of L-AChRs activated by 10 μM ACh in presence of 50 μM thymol were fitted by one exponential component whose mean duration was similar to that of the control condition (Table 1). The mean burst duration was also similar in both conditions (Table 1). Interestingly, at 10 μM ACh the decrease in the frequency of openings due to the presence of 50 μM thymol was not statistically significantly different to that measured at 100 μM ACh (42 ± 24% at 10 μM ACh, n = 7, and 19 ± 9% at 100 μM, n = 5, p>0.3). This finding suggests that thymol is not acting as a competitive antagonist.

Overall, in the presence of three different terpenoids -thymol, carvacrol and eugenol- single-channel activity of L-AChRs is significantly reduced, indicating an inhibitory effect. The results are compatible with action of these drugs as allosteric inhibitors.

## Discussion

Anthelmintic treatment of nematode infections remains the pillar of worm control in both human and veterinary medicine. However, control is threatened by the appearance of drug resistant nematodes, which leads to the need of developing novel compounds.

*C*. *elegans* has been pivotal in anthelmintic drug discovery, in defining mechanisms of action of antiparasitic drugs, and in the understanding of mechanisms of drug resistance [38]. By using *C*. *elegans*, we here defined the anthelmintic actions of three plant terpenoids at the behavioral level, identified muscle L-AChR and GABA receptors as important targets involved in the paralyzing effects, and described the underlying molecular mechanisms. N-AChRs may also participate in terpenoid actions since the mutant worms lacking ACR-16 are more resistant than wild-type worms but this occurs only at low terpenoid concentrations. Interestingly, *Ancylostoma caninum* ACR-16 has been recently proposed as a valid target site for the development of anthelmintics against hookworm infections [39].

Considering previous *in vivo* and *in vitro* studies on parasitic nematodes reporting the efficacy of these terpenoids as antiparasitic drugs [14], our results are of high relevance for their application in anthelminthic therapies.

We found that terpenoids induce *C*. *elegans* paralysis as a function of time and concentration. The rank order of potencies established from the EC50 values for the paralyzing effects are carvacrol > thymol > eugenol, in accordance with previous reports in *Haemonchus contortus* and *C*. *elegans* [9, 28]. We showed that terpenoids produce “isometric” paralysis, in which worm body length does not change significantly during exposure on agar plates, as evaluated by visual inspection and measurement of worm length. On the contrary, the paralysis produced by agonists of L-AChRs, such as levamisole, or agonists of UNC-49 receptors, such as piperazine, is spastic or flaccid, respectively [5,40]. The type of phenotypic changes induced by terpenoids may be due to their dual effects on muscle receptors that mediate antagonistic actions since L-AChRs are involved in muscle contraction and UNC-49 receptors, in muscle relaxation. It is important to note that, although we can ensure that L-AChRs and UNC-49 receptors are markedly involved in the terpenoid paralyzing effects and that N-AChRs may also participate, additional targets -not here explored- could not be discarded. In fact, *C*. *elegans* contains 29 nAChR subunits, which would be worthy of further study to explore terpenoid selectivity in the nicotinic family. Moreover, terpenoids act at receptors of different families, such as transient receptor potential channels [41] that are also present in *C*. *elegans* [42].

Studies on *Ascaris suum* showed that carvacrol inhibits ACh-induced muscle contraction and membrane depolarization [3]. This is in full agreement with our results that demonstrate allosteric inhibition of L-AChR responses to ACh and, therefore, validate *C*. *elegans* as a nematode model for deciphering terpenoid action.

We determined that the sensitivity to terpenoids varies among developmental stages and L1 worms are more sensitive than adult worms. These findings are relevant due to importance of larvae in nematode transmission infections. One possible explanation for the different sensitivity could be due to differences in cuticle permeability between L1 and adult worms [43,44].

The ability to reliably detect anthelmintic resistance is a crucial aspect of resistance management [45]. In this regard, the egg hatch assay has been used as a measurement of drug resistance [29]. In addition to the rapid paralyzing effects mediated by L-AChR and UNC-49 receptors at the neuromuscular junctions, we found that thymol, carvacrol and eugenol increase the fraction of unhatched eggs. This effect would, in turn, lead to a broader anthelmintic spectrum activity. The mode of action by which terpenoids produce egg-hatching inhibition has not been determined yet. Nevertheless, our findings indicate that by two temporarily different mechanisms -rapid paralysis and reduced egg-hatching- terpenoids may have a profound anthelmintic activity. Importantly, similar findings have been reported in the parasitic nematode *Haemonchus contortus* exposed to plant essential oils, which showed ovicidal, larvicide and adulticide effects [8,9, 46].

The combinatorial chemotherapy strategy has been successful in achieving improved efficacy, decreased toxicity, and reduced development of drug resistance for several pathologies, including parasitosis [47]. The use of bioactive natural products as complementary tools to existing synthetic anthelmintic drugs has shown promising results [47]. We showed that combinations of thymol-classic anthelmintics should be carefully evaluated since nematode susceptibility could be changed with respect to that of individual drug effects. At the tested concentrations, thymol-levamisole combinations were more effective to paralyze worms than thymol alone but the combinations thymol-piperazine and thymol-ivermectin were less effective. We determined by the Chou-Talalay’s CI theorem included in Compusyn software that the combination effect is synergic (CI<1) for thymol-levamisole, at least at the tested concentrations. This is a widely used quantitative method that allows determination of synergism, antagonism or additive *in vitro*, in animals and in clinical trials [27,37].

We performed electrophysiological recordings on *C*. *elegans* L1 muscle cells for defining drug action. We measured in the whole-cell configuration responses of L-AChRs and UNC-49 receptors to the terpenoids as well as to their endogenous agonists before and after terpenoid application. Unfortunately, and as reported previously, these cultured muscle cells are not technically suitable for successive drug applications, and we were therefore not able to obtain a complete pharmacological characterization [17,21]. In addition, terpenoids produce instability of membrane patches and high concentrations could not be tested. Nevertheless, we revealed that terpenoids do not activate the receptors and that peak currents elicited by ACh and GABA are reduced by thymol, carvacrol and eugenol, indicating that terpenoids act as inhibitors of both L-AChRs and UNC-49 receptors from *C*. *elegans*.

To further decipher the underlying mechanism of the macroscopic inhibitory effect, we recorded single-channel currents activated by ACh and levamisole that we have previously shown that correspond to the L-AChR [17]. The analysis revealed changes in the activity pattern without changes in amplitude and mean open and burst durations. The main change is evidenced as a marked reduction in the frequency of single L-AChR channel opening, which explains the mechanistic basis of the inhibitory effect. This effect could be due to terpenoid actions as competitive antagonists or negative allosteric inhibitors of L-AChRs. However, since the decrease in the frequency of opening events is similar at a 10-fold change in ACh concentration (10 and 100 μM), the results suggest that terpenoids are allosteric, non-competitive, inhibitors. Molecular mechanisms underlying non-competitive inhibition may be increased desensitization of resting receptors, slow open-channel blockade or impaired opening; these mechanisms cannot be distinguished from our single-channel recordings.

Terpenoids have been shown to have different modulatory actions (positive and negative) at different Cys-loop receptors. For example, thymol is a positive allosteric modulator of the human GABA(A) receptor and of a homo-oligomeric GABA-activated RDL receptor from *Drosophila melanogaster* [48,49]. In the *Drosophila* RDL receptor, substitution of a threonine at 6´position of M2 to methionine, which is found in the mite *Varroa Destructor* RDL receptor, converts thymol into a negative modulator [50]. Monoterpenoids, including thymol, carvacrol and eugenol, act as negative allosteric modulators of α7 nAChR [51]. Thymol and carvacrol display allosteric agonist activity on the human 5-HT_3_A receptor but not on the closely related mouse 5-HT_3_A receptor [52]. Thymol inhibits macroscopic currents of the glutamate-gated chloride channels (GluClRs) from *S*. *mansoni* (SmGluCl-2) and *Haemonchus contortus* (AVR-14B GluCl) [53,54]. The fact that in our assays the triple mutant strain resistant to ivermectin was sensitive to thymol may indicate that for the anthelmintic action either the enhancement of GluClR activity is more important than its inhibition or the effects through L-AChR and GABA receptors are dominant. Docking studies using an NMR structure of the human α7 nAChR transmembrane domain (PDB code 2MAW) [55] indicated that cyclic monoterpenes may interact with an allosteric site located in the transmembrane domain [51]. The authors suggested that the binding of monoterpenes to the closed conformation will prevent the M2 helix from adopting the orientation that is characteristic of the open channel. This interpretation agrees with our results showing reduced frequency of opening events. The action of thymol through a transmembrane domain is also in line with results from mutant RDL receptors and from ELIC-5HT_3_A chimera [50].

The fact that terpenoids act at different nematode receptors has important advantages since diverse lines of evidence indicate that polypharmacological agents, which are those that act simultaneously at various protein targets, might show better profiles than selective ligands, regarding both efficacy and side effects [56]. In addition, by acting at different receptors, terpenoids may reduce the development of resistance since one type of receptor may overcome the decreased drug sensitivity associated to mutations in the other receptor.

Overall, our work indicates that the treatment with terpenoids may be explored as an alternative or complementary anthelmintic strategy, which may help to overcome the ever-increasing resistance of parasites to classical anthelmintic drugs, as well as a supplement to reduce infection burdens of soil transmitted helminths.

## Supporting information

S1 Fig Deciphering the molecular targets mediating terpenoid effects by using mutant strains. (DOCX)

S2 Fig Analysis of the paralyzing effects of thymol, levamisole and thymol/levamisole combinations. The analysis was performed using CompuSyn Software.
(DOCX)
