# Supplementary material for: Caenorhabditis elegans muscle Cys-loop receptors as novel targets of terpenoids with potential anthelmintic activity
Source: PLoS Negl Trop Dis. 2019 Nov 25;13(11):e0007895. doi: 10.1371/journal.pntd.0007895 (PMC6901230; doi:10.1371/journal.pntd.0007895)
Supplement: S1 Fig — (DOCX) [file pntd.0007895.s001.docx]

**Supplementary Figure S1**

**
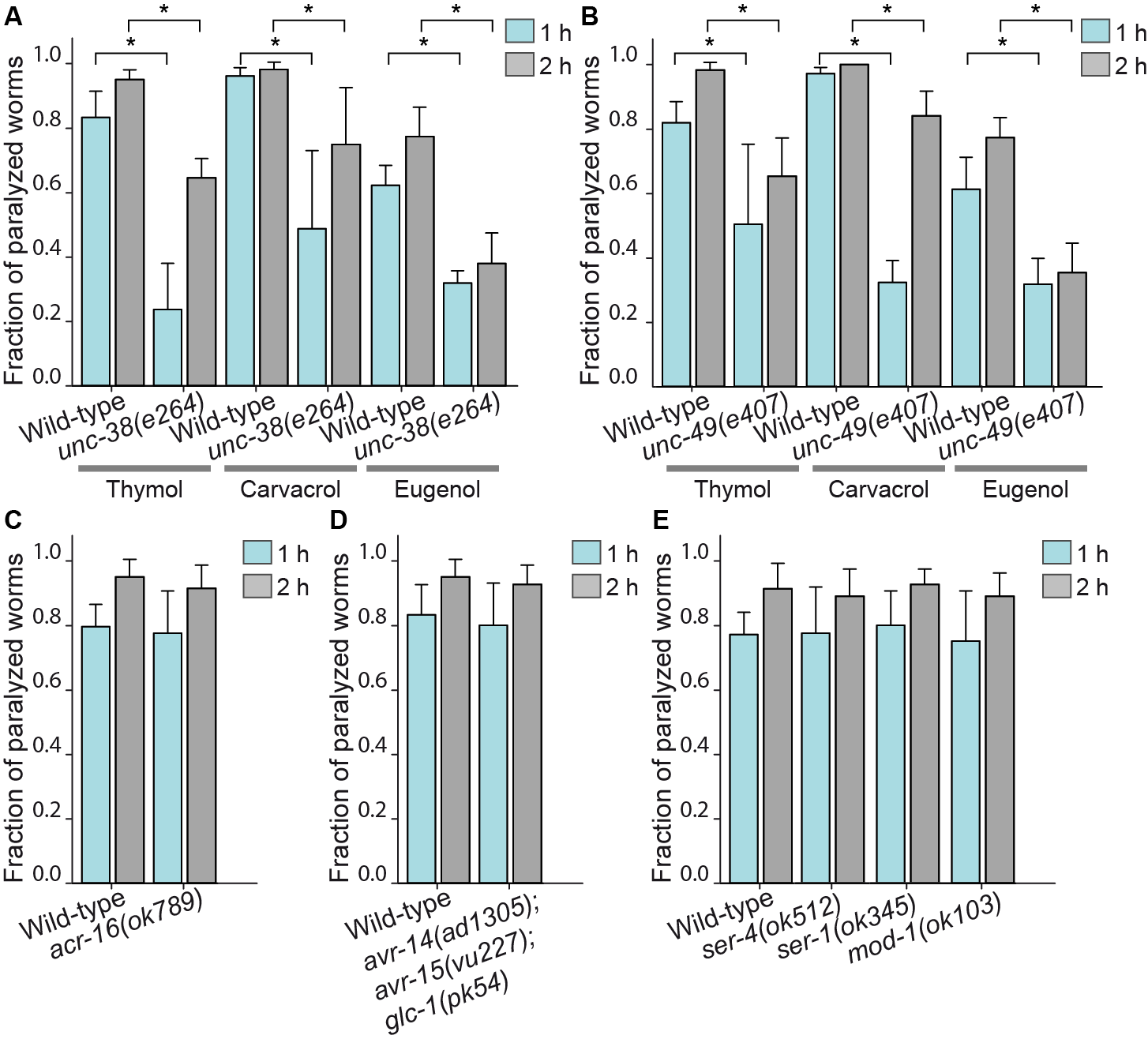
**

***Fig. S1. Deciphering the molecular targets mediating terpenoid effects by using mutant strains.***

Wild-type and mutant adult worms were placed on agar plates containing 1.2 mM thymol, 1.2 mM carvacrol or 1.6 mM eugenol and the fraction of paralyzed worms was measured for each condition. Each point represents the average of 5 experiments, n = 30 worms, error bars = SD. *C. elegans* mutant strains are: (A) *unc-38(e264)*; corresponds to mutants of UNC-38 subunit, which lack functional L-AChRs; (B) *unc-49(e407)*, corresponds to null mutants of UNC-49B subunit, which lack UNC-49 receptor; (C) *acr-16(ok789)* worms that lack the homopentameric nicotine-sensitive AChR (N-AChR or ACR-16); thymol concentration: 1.2 mM; (D) The triple mutant strain of glutamate-gated chloride channel receptor (GluClRs) subunits, *avr-14(ad1305);avr-15(vu227);glc-1(pk54)*, which has been shown to exhibit high resistance to ivermectin; thymol concentration: 1.2 mM; (E) *ser-4(ok512)* that lacks a metabotropic serotonin receptor (SER-4); *ser-1(ok345)* that lacks a metabotropic serotonin receptor (SER-1), and *mod-1(ok103)* that lacks the serotonin-activated chloride channel (MOD-1). Worms were exposed to 1.2 mM thymol.

Results are shown as mean ± S.D. (* p<0,05). The symbol * indicates statistically significant differences between wild-type and mutant worms of the same group (1 h or 2 h exposure).
