# Supplementary material for: Caenorhabditis elegans muscle Cys-loop receptors as novel targets of terpenoids with potential anthelmintic activity
Source: PLoS Negl Trop Dis. 2019 Nov 25;13(11):e0007895. doi: 10.1371/journal.pntd.0007895 (PMC6901230; doi:10.1371/journal.pntd.0007895)
Supplement: S2 Fig — The analysis was performed using CompuSyn Software. (DOCX) [file pntd.0007895.s002.docx]

**Supplementary Figure S2**

**
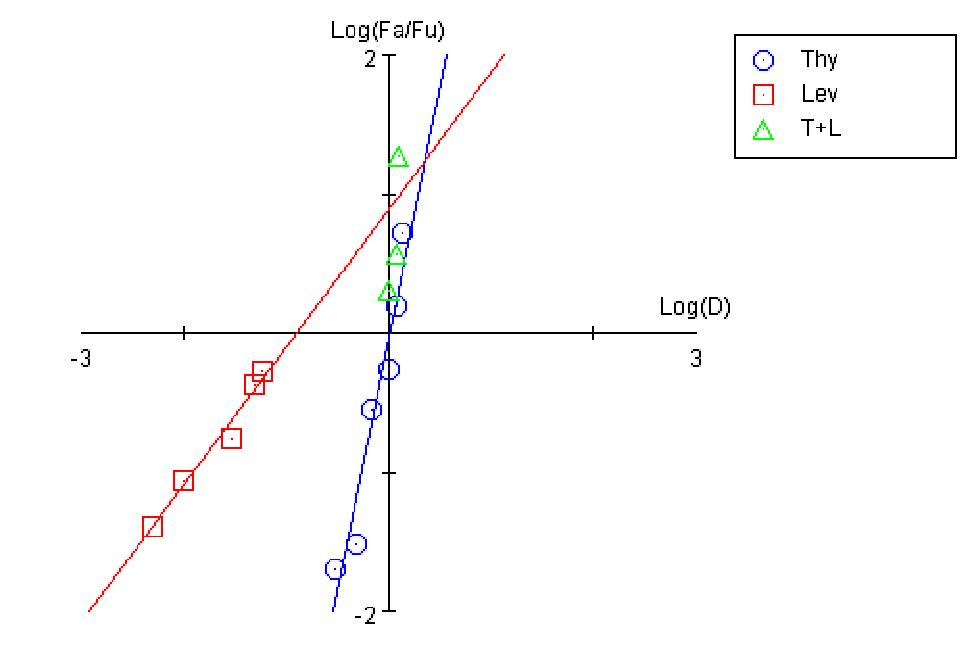
**

**Median-Effect Plot determined by CompuSyn** Software and CI Data for Non-Constant Combo: Thymol (Thy), Levamisole (Lev) and Thymol plus Levamisole (T+L)

Dose Thy Dose Lev Effect CI

1.0 0.03 0.67 0.90486

1.2 0.03 0.79 0.86032

1.2 0.06 0.95 0.53348

**
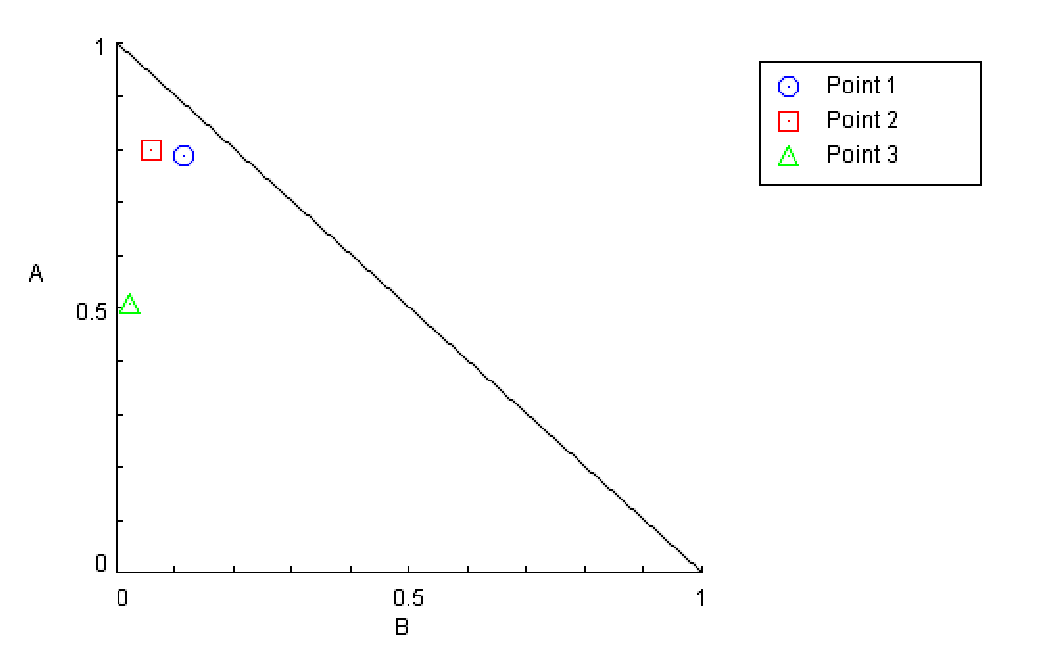
**

**Normalized Isobologram for Combo: Thymol and Levamisole determined by CompuSyn**

Point 1: Combo 1.0 mM Thymol + 0.03 mM Levamisole

Point 2: Combo 1.2 mM Thymol + 0.03 mM Levamisole

Point 3: Combo 1.2 mM Thymol + 0.06 mM Levamisole
